# Association between the triglyceride-glucose index and disease severity in non-diabetic Parkinson’s disease patients

**DOI:** 10.3389/fnagi.2026.1801518

**Published:** 2026-04-29

**Authors:** Deyan Zeng, Min Luo, Baojun Zhang, Yan Zhang, Ailan Pang, Xinglong Yang

**Affiliations:** Department of Neurology, First Affiliated Hospital of Kunming Medical University, Kunming, Yunnan Province, China

**Keywords:** disease severity, insulin resistance, lipid metabolism, non-diabetic Parkinson’s disease patients, triglyceride-glucose index

## Abstract

**Background:**

The triglyceride-glucose (TyG) index is a well-established biomarker of insulin resistance (IR) in non-diabetic populations, serving as a reliable indicator of metabolic health across diverse demographic groups. However, to date, few studies have explored the association between IR—as measured by the TyG index and disease severity in non-diabetic patients with Parkinson’s disease (PD).

**Methods:**

PD Patients were recruited from the Department of Neurology at The First Affiliated Hospital of Kunming Medical University between 2020 and 2025. Disease severity in Parkinson’s disease (PD) was assessed using the Hoehn-Yahr (HY) staging system, where stages I–II correspond to the early phase and stages beyond II indicate intermediate-to-advanced progression. The study compared TyG index differences between these groups. Spearman correlation analysis evaluated associations between individual variables and disease severity in non-diabetic PD patients. Logistic regression analysis calculated odds ratios (ORs) with 95% confidence intervals (CIs), with additional adjustment for PD-specific confounders (disease duration, age at onset, LEDD) and nutritional/metabolic markers (UA, TC, LDL, HDL, creatinine). Restricted cubic spline (RCS) analysis further examined the relationship with disease severity.

**Results:**

A total of 458 non-diabetic patients with Parkinson’s disease (PD) were enrolled. The TyG index was significantly lower in the intermediate-to-advanced stage group compared to the early-stage group (Z = −2.257, *p* = 0.024). Furthermore, univariate and basic multivariate analysis revealed an independent negative association between the TyG index and disease severity (OR = 0.610, 95% CI: 0.380–0.977, *p* = 0.039). After adjusting for PD-specific confounders (disease duration, age at onset, LEDD), regression analysis showed a weakened association between the triglyceride glucose index and disease severity (*β* = −0.445, *p* = 0.057, OR = 0.641, 95%CI: 0.406 ~ 1.013). The association between the triglyceride glucose index and Parkinson’s disease severity was no longer statistically significant after further adjustment for nutritional/metabolic indicators (*β* = −0.179, *p* = 0.598, OR = 0.836, 95%CI: 0.430 ~ 1.625).

**Conclusion:**

In non-diabetic PD patients, a lower TyG index was associated with greater disease severity in more advanced HY stages, but this relationship is not independent after full adjustment. Moreover, reverse causality could not be ruled out and requires validation in future prospective studies.

## 1 Introduction

Parkinson’s disease (PD) is the second most common neurodegenerative disorder worldwide, characterized by both motor and non-motor symptoms. The primary motor manifestations include resting tremor, muscle rigidity, bradykinesia, and impaired gait or postural instability (Rana et al., 2014; Dirkx et al., 2018). However the physiopathologic mechanism of the occurrence and progression of PD still need to be elucidate. Emerging evidence suggests that insulin resistance (IR) contributes to PD progression by disrupting neuronal insulin signaling pathways and promoting *α*-synuclein aggregation (Tan et al., 2024). Notably, approximately 60% of PD patients exhibit IR, as measured by the Homeostatic Model Assessment of Insulin Resistance (HOMA-IR), despite maintaining normoglycemia (Hogg et al., 2018). These findings highlight the potential of antidiabetic agents as promising therapeutic interventions for PD (Nowell et al., 2023), suggesting that targeting IR may slow disease progression and mitigate cognitive decline.

The triglyceride-glucose (TyG) index, calculated from fasting triglyceride and glucose levels, is widely regarded as a reliable surrogate marker for insulin resistance assessment owing to its simplicity, low cost, and high reproducibility. As a reliable biomarker for IR assessment, the TyG index has been extensively utilized in risk stratification for various conditions, including diabetes, cardiovascular and cerebrovascular diseases, and neurodegenerative disorders. A substantial body of evidence has demonstrated a significant association between the TyG index and the incidence, severity, and adverse prognosis of cardiovascular diseases, particularly coronary artery disease and heart failure (Ding et al., 2021; Huang et al., 2022; Liu et al., 2022). In recent years, the potential association between the TyG index and stroke has been increasingly substantiated. Substantial evidence indicates that an elevated TyG index is associated with a higher risk of stroke incidence, recurrence, and adverse clinical outcomes (Wang F. et al., 2022; Zhao et al., 2022; Wang et al., 2025). Associations between the TyG index and cognitive impairment, and dementia have been well documented (Hong et al., 2021; Li et al., 2022; Teng et al., 2022; Tong et al., 2022; Wang K. et al., 2022; Weyman-Vela et al., 2022; Ma et al., 2024). Recent studies further suggest an association between elevated TyG index levels and cognitive impairment in patients with Parkinson’s disease (Cao et al., 2025). However, in these studies, all or a subset of the participants had diabetes.

Previous research has predominantly focused on diabetic populations or broader cohorts that include individuals with diabetes, thereby limiting the generalizability of the findings to non-diabetic populations. To date, no studies have reported an association between disease severity and the TyG index in non-diabetic patients with Parkinson’s disease (PD). Therefore, this retrospective cross-sectional study aims to investigate the association between the TyG index and disease severity in a cohort of 458 non-diabetic PD patients, and to further explore the influence of PD-specific clinical factors and nutritional status on this association.

## 2 Materials and methods

### 2.1 Ethical statement

This retrospective cross-sectional study was conducted in compliance with the ethical principles outlined in the Declaration of Helsinki. The study protocol received formal approval from the Institutional Review Board (IRB) of the First Affiliated Hospital of Kunming Medical University. Written informed consent was obtained from all participants prior to their inclusion in the study. All collected data were anonymized, stored securely, and accessed exclusively for research purposes in accordance with confidentiality protocols.

### 2.2 Study population

Inclusion criteria: (1) Fulfillment of the 2015 International Parkinson and Movement Disorder Society (MDS) clinical diagnostic criteria for Parkinson’s disease. (2) All case evaluations and assessments were independently confirmed by at least two associate chief physicians or higher-ranking specialists to ensure comprehensiveness and accuracy. (3) Fasting blood glucose <7.0 mmol/L, no history of diabetes or use of hypoglycemic medications, and completion of all relevant scale assessments and blood biochemical tests, ensuring complete data availability. (4) Informed consent was obtained from all participants and their family members, and a duly completed clinical informed consent form authorized the use of relevant data for scientific research and statistical analysis. (5) All H-Y staging and UPDRS III assessments were performed in the OFF state (≥12 h after withdrawal of dopaminergic drugs).

Exclusion criteria: (1) Conformity to established exclusion criteria; (2) Clinical diagnosis of secondary parkinsonism, hereditary degenerative Parkinsonian syndromes, or Parkinson-plus syndromes (including progressive supranuclear palsy, dementia with Lewy bodies, multiple system atrophy, Huntington’s disease, etc.); (3) History of stereotactic intracranial surgery (including deep brain stimulation and neuronal nucleus ablation); (4) Presence of significant psychiatric disorders or current use of specific medications, including lipid-lowering agents such as fibrates that may alter serum triglyceride levels; (5) Severe hepatic or renal dysfunction, pre-existing endocrine disorders, or major immunologic diseases; (6) Critical missing clinical data unobtainable through follow-up investigations.

**Figure 1:**
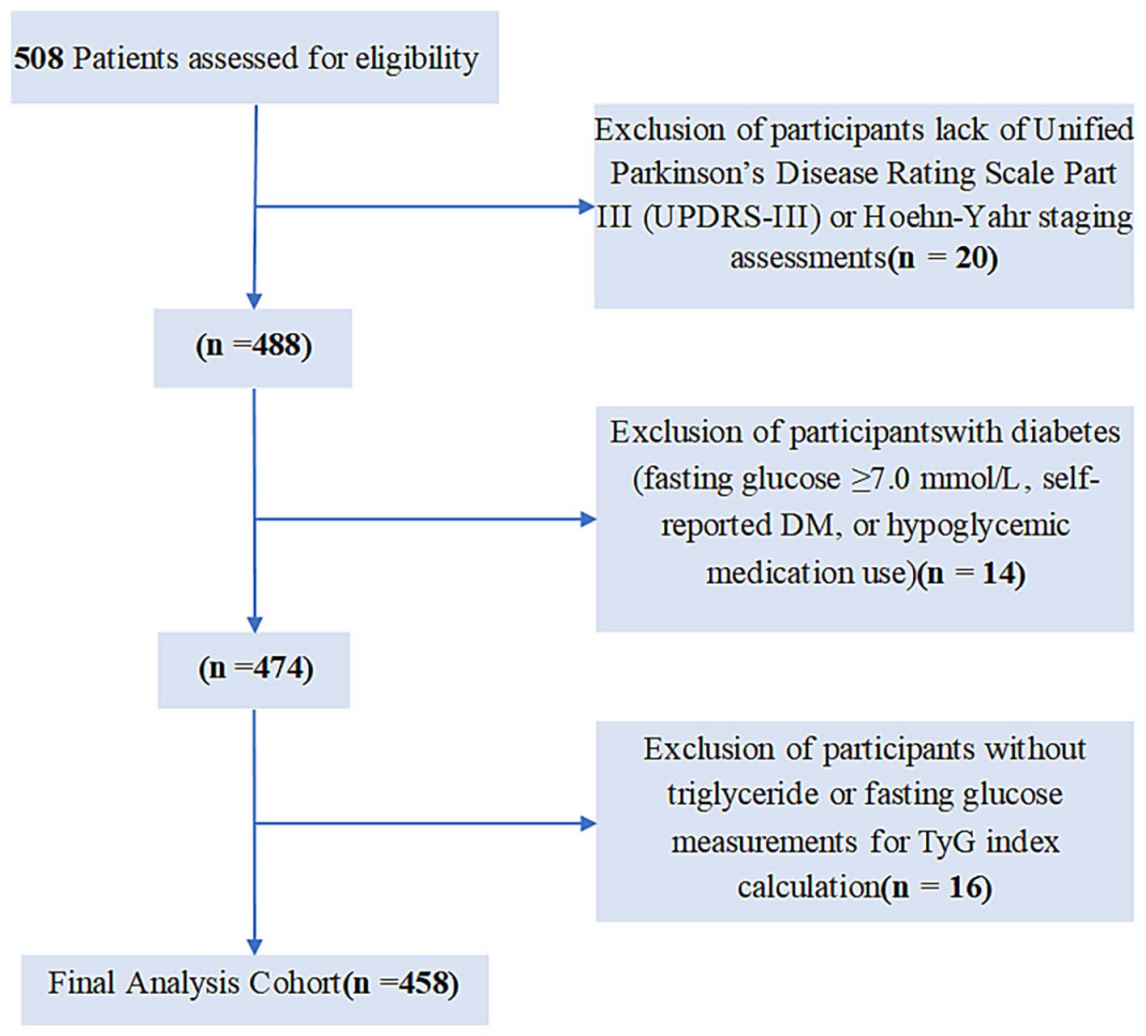
Selection of study population.

A total of 508 patients diagnosed with Parkinson’s disease (including both inpatients and outpatients) at the Second Department of Neurology, First Affiliated Hospital of Kunming Medical University, between January 2020 and January 2025. This study excluded individuals with incomplete UPDRS-III and Hoehn-Yahr staging assessments (*n* = 20). Participants lacking triglyceride and fasting glucose measurements required for TyG index calculation (*n* = 16) were subsequently excluded. Further exclusions applied to subjects with fasting plasma glucose ≥7.0 mmol/L, diagnosed diabetes, or use of hypoglycemic agents or non-fibrate lipid-lowering medications. Diabetes mellitus was defined by self-reported diagnosis, serum fasting glucose ≥126 mg/dL, diabetic history, or hypoglycemic medication use, leading to the exclusion of 14 diabetic patients. Ultimately, 458 participants were included in the final analysis. The detailed selection process for the study cohort is illustrated in Figure 1.

### 2.3 Exposure variables and outcome measures

The TyG index was calculated using the formula: TyG = Ln [serum triglycerides (mg/dL) × glucose (mg/dL)/2] (Liu et al., 2021). The original measurement unit of TG and fasting plasma glucose was mmol/L. Before calculating the TyG index, we converted the unit to mg/dL according to clinical standards. The specific conversion factors are: 1 mmol/L of glucose = 18 mg/dL; 1 mmol/L of TG = 88.57 mg/dL. The TyG index was calculated as ln[TG (mg/dL) × glucose (mg/dL) / 2], which is consistent with the standard calculation method. Due to the mathematical derivation relationship between TG, FPG and the TyG index, TG and FPG were not included in the subsequent regression analysis to avoid multicollinearity.

Disease severity was assessed using the Hoehn and Yahr (HY) staging system (Kataoka and Sugie, 2021), a validated clinical tool for evaluating motor impairment in Parkinson’s disease (PD). The HY scale ranges from 0 (no symptoms) to 5 (severe disability), with stages 1–2 indicating early PD and stages 3–5 representing intermediate to advanced disease. This study adhered to this standardized classification.

Diabetes was defined as: (1) Self-reported physician diagnosis, (2) Use of glucose-lowering medications, (3) Fasting plasma glucose (FPG) ≥ 126 mg/dL (7.0 mmol/L).

Potential confounders included: (1) Demographics: Age, sex, years of education; (2) Anthropometrics: BMI categories (<18.5, 18.5–23.9, ≥24.0–27.9 kg/m^2^); (3) Lifestyle factors: Smoking status (non-smoker = 0, smoker = 1), alcohol consumption (non-drinker = 0, drinker = 1); (4) Comorbidities: Hypertension (absent = 0, present = 1), defined by: (1) Clinical diagnosis. (2) Use of antihypertensive medication. (3) Blood pressure ≥140/90 mmHg. (4) PD-specific clinical factors: disease duration (years), age at onset (years), levodopa equivalent daily dose (LEDD). (5) Nutritional/metabolic proxies: uric acid (UA), total cholesterol (TC), low-density-lipoprotein (LDL), high-density-lipoprotein (HDL), creatinine.

### 2.4 Statistical analysis

All statistical analyses were performed using SPSS 27.0 (IBM Corp., Armonk, NY, USA). Continuous variables with normal distributions were expressed as mean ± standard deviation (x̄ ± SD) and compared using parametric analysis of variance (ANOVA). Non-normally distributed continuous variables were reported as median (interquartile range) [M (Q1, Q3)] and analyzed using the Mann–Whitney U test, preceded by homogeneity of variance testing. Categorical variables were presented as frequencies (percentages) [*n* (%)] and compared using the χ^2^ test.

To examine relationships between independent variables (including the TyG index, age, body mass index [BMI], education duration, smoking history, alcohol consumption, hypertension status, PD-specific clinical factors and nutritional proxies)and disease severity, Spearman correlation analyses were conducted. Correlation heatmaps were generated to visualize multivariate association patterns. Additionally, a restricted cubic spline (RCS) model was employed to assess potential nonlinear dose–response relationships between the TyG index and disease severity. There fore univariate and multivariate binary logistic regression analyses were employed in this study, with three binary logistic regression models established: (1) Unadjusted model; (2) Model adjusted for PD-specific confounders (disease duration, age at onset, LEDD); (3) Model adjusted for PD-specific confounders + nutritional/metabolic markers (UA, TC, LDL, HDL, creatinine). This approach enhances the precision of association analysis. The statistical significance level was set at *α* = 0.05, with *p* < 0.05 considered statistically significant.

Handling of missing data: Levodopa equivalent daily dose (LEDD) showed an extremely right-skewed distribution, with a median of 4.5 (interquartile range: 3.0–187.5). This unusual distribution is a common clinical phenomenon, mainly attributed to individual differences in patients’ treatment regimens and disease conditions, which does not affect the reliability and validity of the study results. A total of 39 out of 458 enrolled patients had missing LEDD data, with a missing rate of 8.52% (39/458). To avoid bias from extreme dose values and the impact of missing data on the study results, missing values were imputed using the median LEDD value of the corresponding group.

## 3 Results

### 3.1 Baseline characteristics

This study included 458 non-diabetic patients with Parkinson’s disease (PD), stratified by disease severity using the Hoehn-Yahr (H-Y) staging system in the OFF state: 321 patients (70.1%) were classified as early-stage (H-Y stages 1–2: 174 cases of stage 1, 147 cases of stage 2) and 137 (29.9%) as intermediate-to-advanced stage (H-Y stages 3–5: 85 cases of stage 3, 42 cases of stage 4, 10 cases of stage 5). Baseline demographic and clinical characteristics differed significantly between the two groups (*p* < 0.05). The intermediate-to-advanced stage group had a significantly older age (median, 71.0 [IQR: 63.75–75.25] years) compared with the early-stage group (median, 62.50 [IQR: 54.00–70.00] years), while the sex distribution was comparable between groups (male: 54.545% vs. 55.521%). Significant intergroup differences were observed in body mass index (BMI), triglyceride-glucose (TyG) index, total cholesterol (TC), low-density lipoprotein cholesterol (LDL), uric acid (UA), disease duration, age at onset, H-Y stage, and UPDRS-III score, as well as hypertension prevalence. In contrast, no significant intergroup differences were found in high-density lipoprotein cholesterol (HDL), creatinine, levodopa equivalent daily dose (LEDD), years of education, smoking history, or alcohol consumption (all *p* > 0.05). Baseline characteristics are summarized in Table 1.

**Table 1:** Comparison of demographic and clinical indicators between patients with early-stage and mid-to-late-stage Parkinson’s disease [M (Q_1_, Q_3_), mean ± SD].

Variables	Total (*n* = 458)	Early stage (*n* = 321)	Mid-to-late stage (*n* = 137)	Statistic	*p*
TYG, M (Q_1_, Q_3_)	8.361 (8.084, 8.688)	8.385 (8.107, 8.706)	8.265 (8.000, 8.662)	*Z* = −2.257	**0.024**
Age, M (Q_1_, Q_3_)	65.00 (56.00, 72.00)	62.50 (54.00, 70.00)	71.00 (63.75, 75.25)	*Z* = −6.631	**<0.001**
BMI, Mean ± SD	23.172 ± 3.193	23.393 ± 3.188	22.627 ± 3.152	*t* = 2.337	**0.020**
TC, Mean ± SD	4.420 ± 0.952	4.514 ± 0.970	4.189 ± 0.867	*t* = 3.342	**<0.001**
LDL, Mean ± SD	2.668 ± 0.812	2.769 ± 0.814	2.420 ± 0.753	*t* = 4.237	**<0.001**
HDL, M (Q_1_, Q_3_)	1.260 (1.060, 1.500)	1.240 (1.060, 1.480)	1.325 (1.080, 1.555)	*Z* = −1.746	0.081
UA, M (Q_1_, Q_3_)	315.550 (262.875, 365.100)	322.650 (274.925, 371.175)	299.800 (249.375, 349.600)	*Z* = −3.183	**0.001**
Createinine, M (Q_1_, Q_3_)	74.050 (63.800, 84.725)	75.200 (64.425, 85.475)	72.900 (62.975, 84.500)	*Z* = −0.869	0.385
LEDD, M (Q_1_, Q_3_)	4.500 (3.000, 9.900)	4.500 (3.000, 65.715)	5.000 (3.150, 8.688)	*Z* = −1.407	0.159
Disease duration, M (Q_1_, Q_3_)	3.000 (1.000, 5.000)	2.000 (1.000, 4.000)	3.000 (2.000, 6.000)	*Z* = −4.241	**<0.001**
Age at onset, Mean ± SD	60.478 ± 11.073	58.633 ± 11.061	65.034 ± 9.739	*t* = −5.800	**<0.001**
H-Y, M (Q_1_, Q_3_)	2.000 (1.000, 3.000)	1.000 (1.000, 2.000)	3.000 (3.000, 4.000)	*Z* = −17.083	**<0.001**
UPDRS III, M (Q_1_, Q_3_)	27.500 (17.000, 42.000)	23.000 (15.000, 33.000)	43.000 (32.000, 56.000)	*Z* = −10.523	**<0.001**
Years of education, M (Q_1_, Q_3_)	9.000 (6.000, 12.000)	9.000 (6.000, 12.000)	7.000 (5.000, 12.000)	*Z* = −1.822	0.068
Sex, *n* (%)				*χ*^2^ = 0.036	0.849
Female	205 (44.760)	145 (44.479)	60 (45.455)		
Male	253 (55.240)	181 (55.521)	72 (54.545)		
Hypertension, *n* (%)				*χ*^2^ = 4.986	0.026
No	306 (66.812)	228 (69.939)	78 (59.091)		
Yes	152 (33.188)	98 (30.061)	54 (40.909)		
Smoking, *n* (%)				*χ*^2^ = 1.583	0.208
No	391 (85.371)	274 (84.049)	117 (88.636)		
Yes	67 (14.629)	52 (15.951)	15 (11.364)		
Alcohol, *n* (%)				*χ*^2^ = 0.140	0.708
No	409 (89.301)	290 (88.957)	119 (90.152)		
Yes	49 (10.699)	36 (11.043)	13 (9.848)		

Mann–Whitney U test was used for inter-group comparison; UPDRS III = Unified Parkinson’s Disease Rating Scale (Motor Function, assessed in OFF state); H-Y Stage = Hoehn-Yahr Stage of Parkinson’s Disease (assessed in OFF state); BMI = Body Mass Index; TYG = Triglyceride-Glucose Index; LEDD = levodopa equivalent daily dose; Z: Mann–Whitney test, *χ*^2^: Chi-square test, M: Median, Q_1_: 1st Quartile, Q_3_: 3rd Quartile. Bold values indicate statistically significant differences (two-sided *p* < 0.05).

### 3.2 Association between triglyceride-glucose index and disease severity

**Figure 2:**
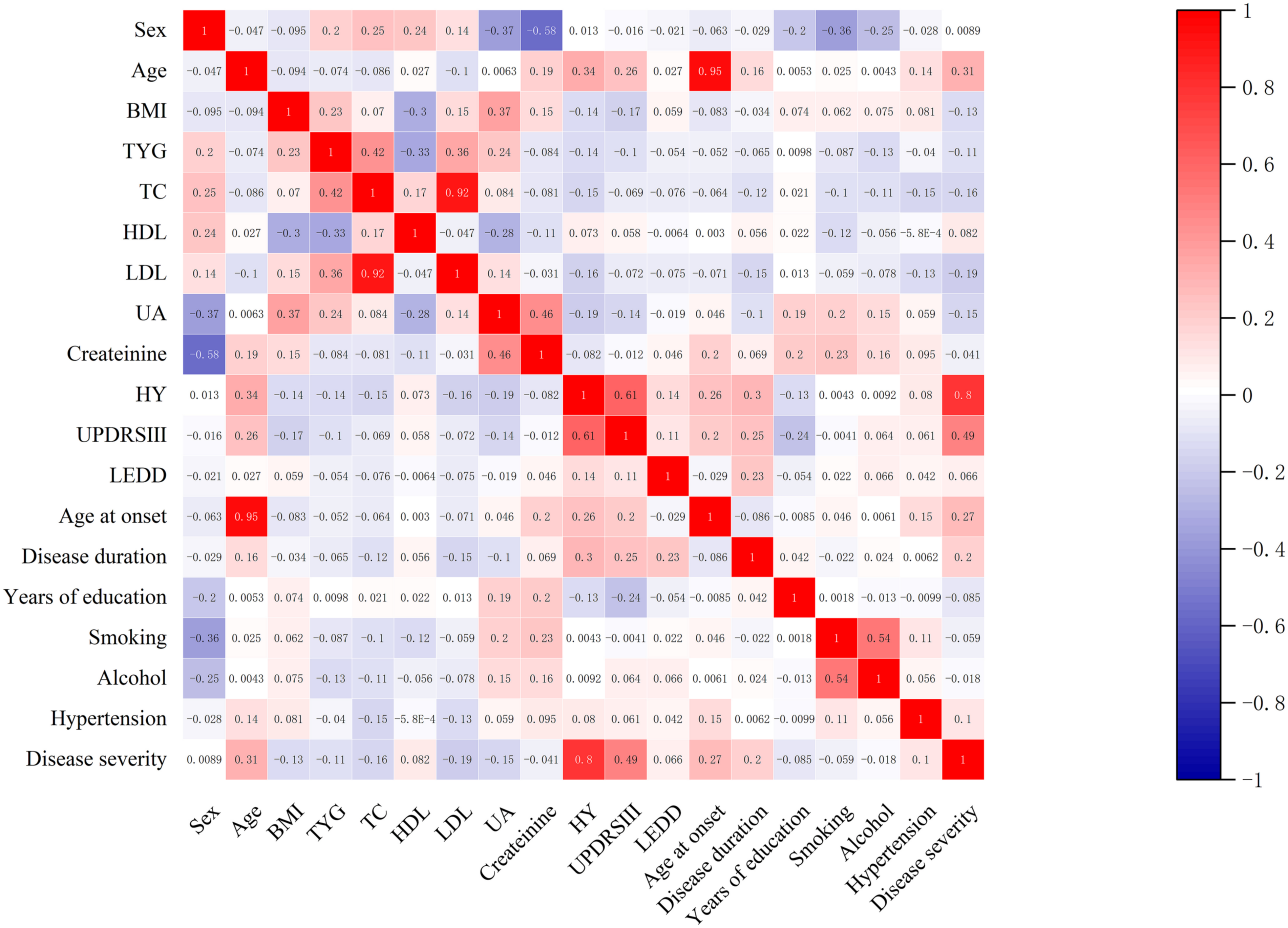
Heatmap of correlation between triglyceride-glucose index and disease severity.

To assess the relationships between disease severity and independent variables (age, sex, education duration, BMI, smoking status, alcohol consumption, hypertension, PD-specific clinical factors and nutritional markers), we performed correlation analyses. Heatmaps were generated to visualize these associations (Figure 2). The analysis revealed a strong positive correlation between disease severity and both Hoehn-Yahr (H-Y) stage (*r* = 0.80, *p* < 0.001) and Unified Parkinson’s Disease Rating Scale Part III (UPDRS-III) scores (*r* = 0.49, *p* < 0.001). Additionally, H-Y stage and UPDRS-III scores were positively correlated (*r* = 0.61, *p* < 0.001). The triglyceride-glucose (TyG) index showed a near-perfect positive correlation with triglyceride (TG) levels (*r* = 0.94, *p* < 0.001) but a weak, statistically significant negative correlation with disease severity (*r* = −0.11, *p* = 0.02). Disease duration and age at onset were positively correlated with PD severity (*r* = 0.20, *p* < 0.001; *r* = 0.35, *p* < 0.001), and UA, TC, LDL were negatively correlated with PD severity (all *p* < 0.05). Other variables exhibited varying degrees of association with disease severity and each other, with correlation coefficients ranging from weakly positive to negative, underscoring the multifactorial nature of PD progression.

### 3.3 Association between the triglyceride-glucose index and disease severity in non-diabetic Parkinson’s disease patients

**Figure 3:**
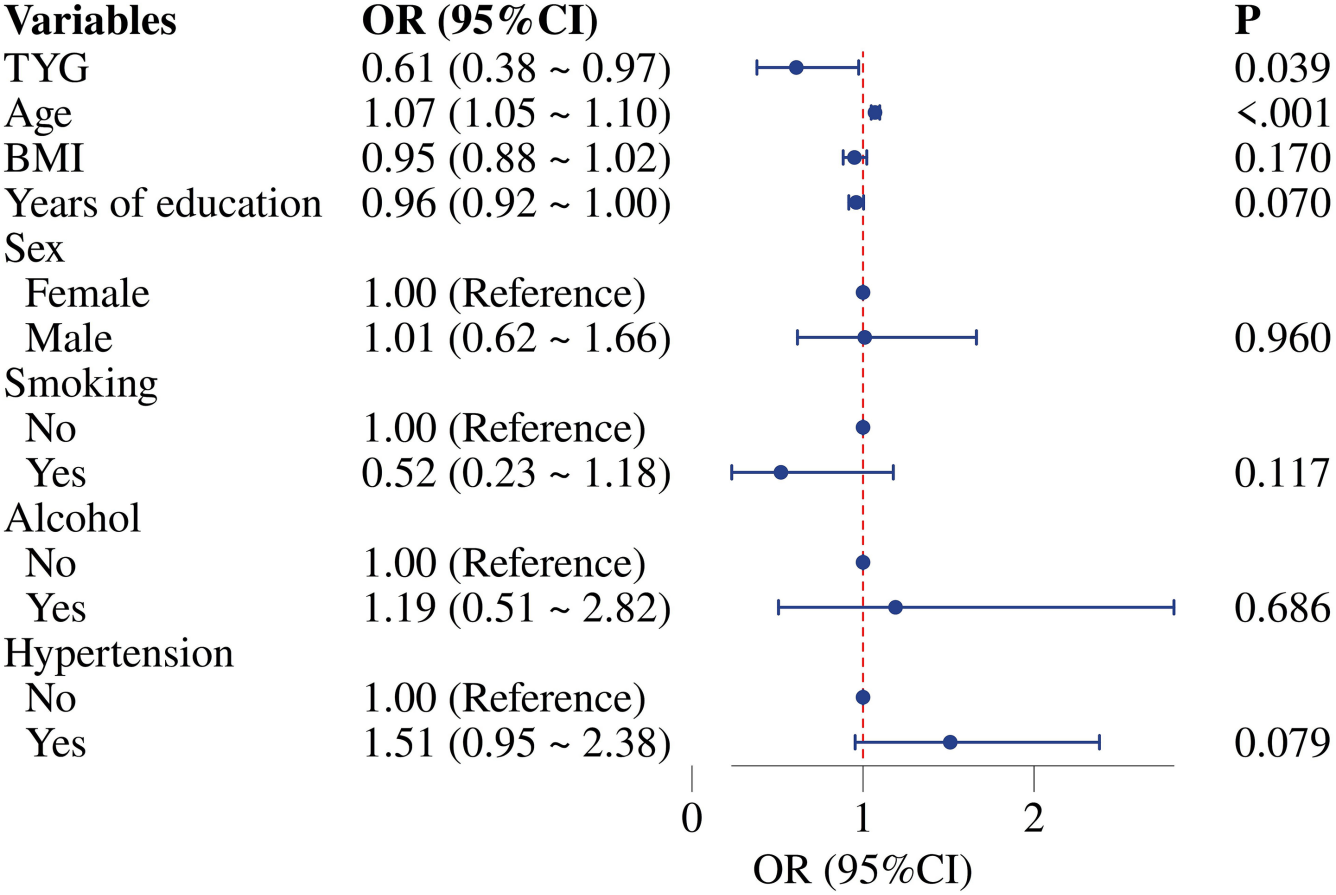
Forest plot of multivariate logistic regression analysis.

To examine the relationship between the triglyceride-glucose (TyG) index and disease severity in non-diabetic Parkinson’s disease (PD) patients, we performed univariate and multivariate logistic regression analyses. In the univariate analysis, the TyG index (OR = 0.560, 95% CI: 0.365–0.859, *p* = 0.008), body mass index (BMI; OR = 0.926, 95% CI: 0.867 ~ 0.988, *p* = 0.021), hypertension (OR = 1.611, 95% CI: 1.058 ~ 2.451, *p* = 0.026), and age (OR = 1.074, 95% CI: 1.050 ~ 1.099, *p* < 0.001) were significantly associated with disease severity (Table 2). After adjusting for potential confounders in the multivariate model, the TyG index (OR = 0.608, 95% CI: 0.380 ~ 0.974, *p* = 0.039) and age (OR = 1.073, 95% CI: 1.048 ~ 1.098, *p* < 0.001) remained significant predictors of disease severity. In contrast, BMI, sex, hypertension, smoking status, alcohol consumption, and years of education showed no statistically significant associations in the multivariate analysis (Table 2; Figure 3).

**Table 2:** Univariate and multivariate logistic regression analyses of the association between triglyceride-glucose index and disease severity in non-diabetic Parkinson’s disease patients.

Variables	Univariate analysis			Multivariate analysis		
	β	*p*	OR (95%CI)	β	*p*	OR (95%CI)
TYG	−0.580	**0.008**	0.560 (0.365 ~ 0.859)	−0.497	**0.039**	0.608 (0.380 ~ 0.974)
Age	0.072	**<0.001**	1.074 (1.050 ~ 1.099)	0.070	**<0.001**	1.073 (1.048 ~ 1.098)
BMI	−0.077	**0.021**	0.926 (0.867 ~ 0.988)	−0.051	0.170	0.950 (0.884 ~ 1.022)
Years of education	−0.037	0.077	0.964 (0.926 ~ 1.004)	−0.042	0.070	0.959 (0.917 ~ 1.003)
Sex						
No			1.000 (Reference)			1.000 (Reference)
Yes	0.039	0.849	1.040 (0.693 ~ 1.562)	−0.013	0.960	0.987 (0.601 ~ 1.622)
Smoking						
No			1.000 (Reference)			1.000 (Reference)
Yes	−0.392	0.210	0.676 (0.366 ~ 1.248)	−0.648	0.117	0.523 (0.232 ~ 1.177)
Alcohol						
No			1.000 (Reference)			1.000 (Reference)
Yes	−0.128	0.708	0.880 (0.451 ~ 1.718)	0.177	0.686	1.194 (0.506 ~ 2.818)
Hypertension						
No			1.000 (Reference)			1.000 (Reference)
Yes	0.477	**0.026**	1.611 (1.058 ~ 2.451)	0.411	0.079	1.508 (0.954 ~ 2.383)

Univariate logistic regression was used to preliminarily screen variables associated with disease severity, while multivariate logistic regression was used to validate associations after adjusting for confounding factors. OR = odds ratio, CI = confidence interval; “0” indicates the reference group for each categorical variable; *p* < 0.05 denotes a statistically significant difference. Bold values indicate statistically significant differences (two-sided *p* < 0.05).

### 3.4 Dose–response relationship between TyG index and disease severity

**Figure 4:**
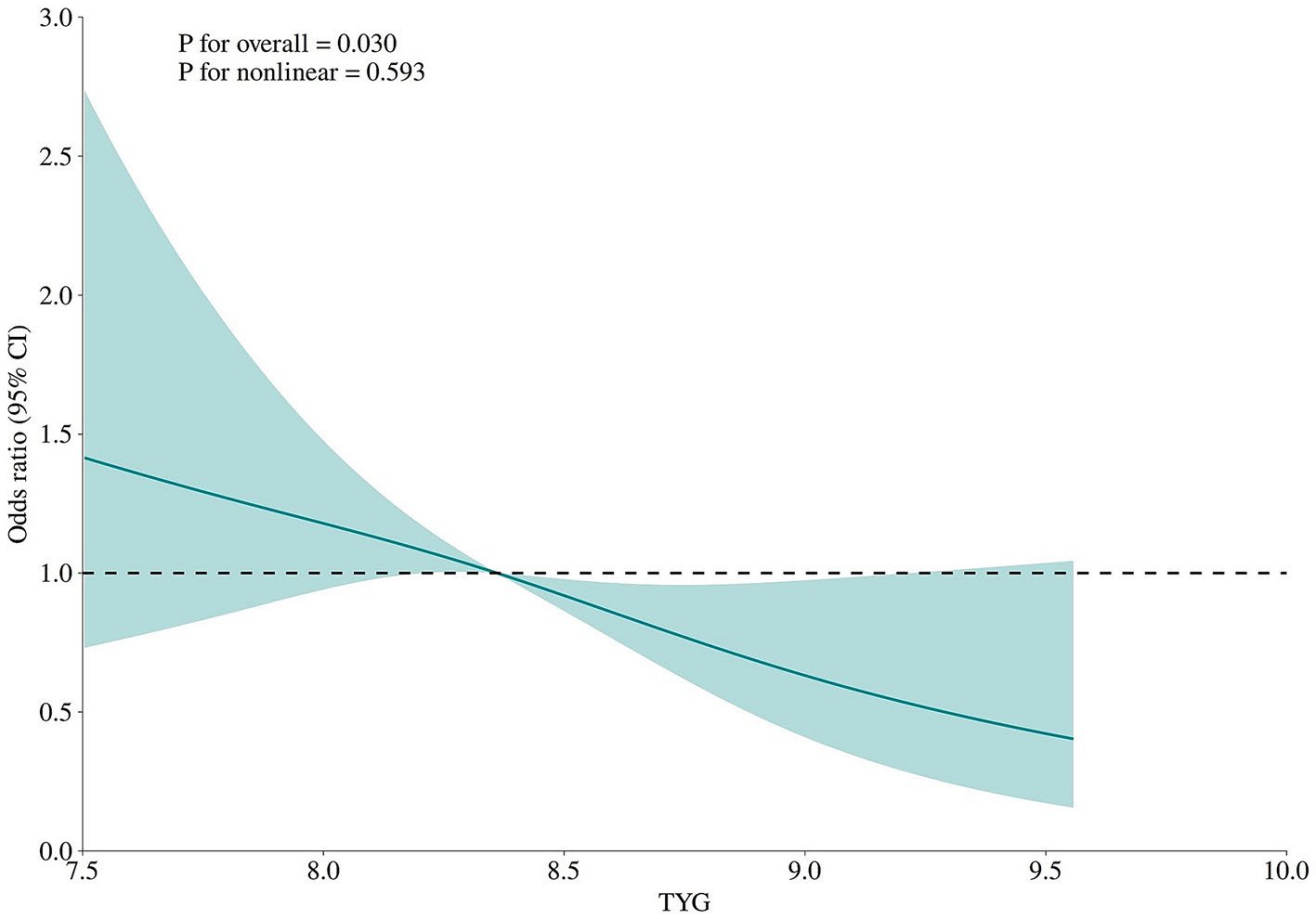
Restricted cubic spline plot depicting the dose–response relationship between triglyceride-glucose (TyG) index and disease severity. TyG index (x-axis) vs. severe disease odds ratio (y-axis; 95%CI = shaded area). Significant overall association (*p* = 0.030); no nonlinearity (*p* = 0.593): higher TyG lower severe disease odds.

To further explore the dose–response relationship between the TyG index and disease severity, we employed a restricted cubic spline (RCS) model. The analysis revealed a significant dose–response association (*p* = 0.030), with a nonlinear *p*-value of 0.593, indicating an approximately linear trend. Specifically, when the TyG index was below 8.5, the odds ratio (OR) exceeded 1, suggesting that lower TyG levels were associated with increased disease severity. Conversely, when the TyG index exceeded 8.5, the OR fell below 1, implying that higher TyG levels correlated with reduced disease severity (Figure 4).

### 3.5 Multivariate logistic regression results in sequentially adjusted models

In this study, stepwise adjusted binary logistic regression was employed, with three models constructed sequentially: an unadjusted model (Table 3), a model adjusted for core confounders specific to Parkinson’s disease (Table 4), and a fully adjusted model further adjusted for nutritional and metabolic indicators (Table 5).

**Table 3:** Unadjusted model.

Variables	β	*p*	OR (95%CI)
TYG	−0.580	**0.008**	0.560 (0.365 ~ 0.859)
Age	0.072	**<0.001**	1.074 (1.050 ~ 1.099)
BMI	−0.077	**0.021**	0.926 (0.867 ~ 0.988)
UA	−0.004	**0.001**	0.996 (0.993 ~ 0.998)
TC	−0.376	**0.001**	0.687 (0.548 ~ 0.861)
HDL	0.644	0.050	1.903 (0.999 ~ 3.626)
LDL	−0.568	**<0.001**	0.566 (0.431 ~ 0.744)
Createinine	−0.004	0.585	0.996 (0.984 ~ 1.009)
LEDD	−0.000	0.498	1.000 (0.998 ~ 1.001)
Age at onset	0.059	**<0.001**	1.060 (1.038 ~ 1.083)
Disease duration	0.102	**0.002**	1.107 (1.038 ~ 1.181)
Years of education	−0.037	0.077	0.964 (0.926 ~ 1.004)
Sex			
Female			1.000 (Reference)
Male	−0.039	0.849	0.961 (0.640 ~ 1.443)
Smoking			
No			1.000 (Reference)
Yes	−0.392	0.210	0.676 (0.366 ~ 1.248)
Alcohol			
No			1.000 (Reference)
Yes	−0.128	0.708	0.880 (0.451 ~ 1.718)
Hypertension			
No			1.000 (Reference)
Yes	0.477	**0.026**	1.611 (1.058 ~ 2.451)

Bold values indicate statistically significant differences (two-sided *p* < 0.05).

**Table 4:** Model adjusted for PD-specific confounders.

Variables	Univariate analysis			Multivariate analysis		
	β	*p*	OR (95%CI)	β	*p*	OR (95%CI)
TYG	−0.580	**0.008**	0.560 (0.365 ~ 0.859)	−0.445	0.057	0.641 (0.406 ~ 1.013)
Age	0.072	**<0.001**	1.074 (1.050 ~ 1.099)	0.677	0.202	1.969 (0.695 ~ 5.578)
BMI	−0.077	**0.021**	0.926 (0.867 ~ 0.988)	−0.062	0.095	0.940 (0.874 ~ 1.011)
Age at onset	0.059	**<0.001**	1.060 (1.038 ~ 1.083)	−0.610	0.250	0.543 (0.192 ~ 1.537)
Disease duration	0.102	**0.002**	1.107 (1.038 ~ 1.181)	−0.532	0.319	0.588 (0.206 ~ 1.674)
LEDD	−0.000	0.498	1.000 (0.998 ~ 1.001)	−0.001	0.146	0.999 (0.998 ~ 1.000)
Hypertension						
No			1.000 (Reference)			1.000 (Reference)
Yes	0.477	**0.026**	1.611 (1.058 ~ 2.451)	0.383	0.101	1.466 (0.928 ~ 2.318)

Bold values indicate statistically significant differences (two-sided *p* < 0.05).

**Table 5:** Model adjusted for PD-specific confounders + nutritional markers.

Variables	Univariate analysis			Multivariate analysis		
	β	*p*	OR (95%CI)	β	*p*	OR (95%CI)
TYG	−0.580	**0.008**	0.560 (0.365 ~ 0.859)	−0.179	0.598	0.836 (0.430 ~ 1.625)
Age	0.072	**<0.001**	1.074 (1.050 ~ 1.099)	0.613	0.271	1.846 (0.620 ~ 5.500)
BMI	−0.077	**0.021**	0.926 (0.867 ~ 0.988)	−0.026	0.515	0.974 (0.901 ~ 1.053)
UA	−0.004	**0.001**	0.996 (0.993 ~ 0.998)	−0.003	0.052	0.997 (0.993 ~ 1.000)
TC	−0.376	**0.001**	0.687 (0.548 ~ 0.861)	0.205	0.645	1.228 (0.512 ~ 2.942)
HDL	0.644	0.050	1.903 (0.999 ~ 3.626)	−0.020	0.972	0.981 (0.326 ~ 2.947)
LDL	−0.568	**<0.001**	0.566 (0.431 ~ 0.744)	−0.639	0.171	0.528 (0.212 ~ 1.317)
Age at onset	0.059	**<0.001**	1.060 (1.038 ~ 1.083)	−0.541	0.331	0.582 (0.196 ~ 1.733)
Disease duration	0.102	**0.002**	1.107 (1.038 ~ 1.181)	−0.483	0.389	0.617 (0.206 ~ 1.850)
LEDD	−0.000	0.498	1.000 (0.998 ~ 1.001)	−0.001	0.165	0.999 (0.998 ~ 1.000)
Createinine	−0.004	0.585	0.996 (0.984 ~ 1.009)	−0.007	0.405	0.993 (0.977 ~ 1.009)
Hypertension						
No			1.000 (Reference)			1.000 (Reference)
Yes	0.477	**0.026**	1.611 (1.058 ~ 2.451)	0.362	0.135	1.436 (0.894 ~ 2.308)

Bold values indicate statistically significant differences (two-sided *p* < 0.05).

In the unadjusted model, which exclusively examined the crude association between the TyG index (exposure) and PD severity (outcome) without adjustment for any confounders, a statistically significant negative association was detected (OR = 0.560, 95% CI: 0.365–0.859, *p* = 0.008), providing preliminary evidence for a potential inverse correlation. However, given the absence of covariate adjustment, this crude association was vulnerable to confounding bias and could not reflect a true independent relationship. Subsequently, a model adjusted for core PD-specific confounders—including disease duration, age of onset, and levodopa equivalent daily dose (LEDD) was established. Following adjustment, the association between the TyG index and PD severity showed marginal statistical significance (OR = 0.641, 95% CI: 0.460–1.013, *p* = 0.057). The OR value was slightly attenuated toward the null compared with the unadjusted model, suggesting that core PD-related factors (disease progression and therapeutic status) exerted a confounding effect and weakened the inverse association. Notably, the direction of association remained consistent (OR < 1), indicating that the potential inverse relationship was not fully explained by these key clinical variables. In the fully adjusted model, nutritional and metabolic surrogate markers including UA, TC, LDL, HDL, and creatinine were further incorporated to achieve comprehensive confounding control. After full adjustment for both PD core factors and metabolic indices, the association between the TyG index and PD severity was no longer statistically significant (OR = 0.836, 95% CI: 0.430–1.625, *p* = 0.598), with the OR value moving closer to 1. These findings imply that the initial inverse association observed in unadjusted and core confounders specific to Parkinson’s disease adjusted models may be driven by confounding from nutritional, metabolic, and PD-specific clinical factors.

## 4 Discussion

This study investigated the association between TyG index and disease severity in 458 non-diabetic Parkinson’s disease (PD) patients. To our knowledge, this is the first systematic investigation of this association in a non-diabetic PD cohort and providing novel insights into the metabolic correlates of PD severity.

Current literature on the relationship between the TyG index and PD severity in non-diabetic patients remains limited. Chang et al. reported a nonlinear (J-shaped) association between the TyG index and PD risk, particularly in non-diabetic individuals (Chang et al., 2025). Similar nonlinear trends have been observed in other conditions, such as atrial fibrillation and cardiovascular mortality in diabetic patients (Liu et al., 2023; Shi et al., 2025). However, emerging clinical data indicate a decline in the TyG index following PD symptom onset, aligning with our findings of a negative correlation between the TyG index and disease severity, which contrary to its positive association with conditions like ischemic stroke (Yang et al., 2023; Prasad et al., 2025).

Given that the TyG index is derived from triglyceride (TG) and fasting blood glucose (FBG) levels, lipid metabolism and glycometabolism plays a crucial role in its dynamics. In one hand, emerging evidence suggests an association between lipid metabolism and the onset and progression of Parkinson’s disease (PD). A large-scale longitudinal cohort study conducted in Israel, involving individuals not taking statins, reported a PD incidence of 0.3% after an average follow-up of 7.9 years. The study found that elevated serum levels of total cholesterol (TC) and low-density lipoprotein cholesterol (LDL-C) in men were associated with a reduced PD risk (Rozani et al., 2018). Similarly, a Swiss cohort study demonstrated that triglycerides (TG), TC, LDL-C, and apolipoprotein B levels were inversely associated with PD risk, with no significant sex-based differences (Dong et al., 2021). Prospective studies indicate that higher serum triglyceride levels correlate with a decreased risk of future PD (Sääksjärvi et al., 2015; Fang et al., 2019; Jing et al., 2025), while case–control studies report lower TG concentrations in PD patients (Scigliano et al., 2006; Wei et al., 2013). Additionally, one study assessed the relationship between lipid profiles and motor performance in PD, revealing that higher TG levels were associated with better motor function (Zhang et al., 2022). This aligns with our present study where a higher TyG index was associated with less severe PD symptoms. In other hand, elevated FBG (predominantly in the prediabetic range of 5.6–6.9 mmol/L) is closely linked to more severe motor dysfunction and accelerated disease progression (Chang et al., 2025). Longitudinal data from the PPMI cohort further confirms that non-diabetic PD patients with higher baseline TyG index experience faster cognitive decline and greater reduction in striatal dopamine transporter activity over 4 years (Cao et al., 2025). Animal studies using PD models have confirmed that long-term hyperglycemia (mimicking prediabetic states) exacerbates pathological accumulation of *α*-synuclein, leading to more extensive loss of dopaminergic neurons and worsening motor deficits (Sato et al., 2011; Lv et al., 2022). These data seems contrary to our result, however there are plausible explanations: Our study enrolled non-diabetic PD patients whose fasting plasma glucose levels, although below 7 mmol/L, included individuals with impaired fasting glucose (i.e., those in the pre-diabetic range of 5.6–6.9 mmol/L). Consequently, an elevated TyG index was observed even in the early stages of PD.

Meanwhile, compared with patients with early-stage Parkinson’s disease (PD), those with mid-to-late-stage PD are affected by multiple confounding factors including insufficient nutritional intake, reduced muscle mass, and decreased glycogen reserves resulting from disease progression. In addition, dopaminergic treatment (such as LEDD and different medication classes) can further influence appetite, body weight, and metabolism. The results of the three regression models included in this study also suggest that the association between TyG and disease severity is influenced by nutritional, metabolic, and PD-specific clinical factors. The observed lower TyG with higher H-Y may reflect malnutrition/catabolic state, reduced lipid stores, sarcopenia, and reduced intake in advanced PD. Thus, low TyG may as a marker of nutritional/catabolic status in advanced PD.

While a low TyG index typically indicates enhanced insulin sensitivity and favorable lipid profiles, excessively low values could signal catabolic states or advanced disease. This duality underscores the TyG index’s context-dependent utility: elevated values in preclinical studies may predict PD risk, whereas lower values in clinical cohorts may reflect disease progression and treatment effects. For example, Chang et al. identified the TyG index as a PD risk predictor in a South Korean population, while Prasad et al. observed declining TyG values in Indian PD patients, likely due to disease progression and dopaminergic therapy (Chang et al., 2025; Prasad et al., 2025). Longitudinal studies tracking TyG index dynamics across PD stages, with adjustments for treatment and population-specific norms, are needed to validate its dual role as both a risk and progression marker. Such research could inform PD screening, monitoring, and metabolic therapy development. Additionally, no significant relationship was observed between body mass index (BMI) and PD severity in this study. While elevated BMI is a recognized risk factor for metabolic syndrome, it may also reflect greater muscle mass and improved nutritional status, which could confer neuroprotective benefits. For instance, higher BMI has been associated with slower cognitive decline and reduced dementia risk in PD patients (Yoo et al., 2019). In our analysis, BMI initially showed a negative correlation with disease severity in univariate regression (*p* = 0.021), but this association became nonsignificant after adjusting for confounders.

Although some interesting results was found in the present research, several limitaions also exist: Firstly, this study has a retrospective cross-sectional design, which can only illustrate association rather than causality, and reverse causality cannot be completely excluded. Secondly, Because of the retrospective design of this study, the absence of glycated hemoglobin (HbA1c) and homeostatic model assessment of insulin resistance (HOMA-IR) limits deeper metabolic analysis. Thirdly, long-term dietary and nutritional influences were not assessed, possibly affecting TyG index accuracy. Fourthly, no follow-up verification was conducted for patients with H-Y stage 1, so the long-term diagnostic stability of patients at this stage cannot be further verified.

This study demonstrates a association between the TyG index and disease severity in non-diabetic Parkinson’s disease (PD) patients. In conclusion, in non-diabetic PD patients, a lower TyG index was significantly associated with greater disease severity. However, reverse causality cannot be excluded in this study and it need to be verified in future perspective study.

## Data Availability

The original contributions presented in the study are included in the article/supplementary material, further inquiries can be directed to the corresponding author.
